# Mesenteric Defects Closure Using N-Hexyl-Cyanoacrylate Surgical Glue During Roux-en-Y Gastric Bypass: A Single-Center Observational Study

**DOI:** 10.7759/cureus.108009

**Published:** 2026-04-30

**Authors:** Hashem Moazenzadeh, Giorgio Badessi, Yaacov Jaoui, Linda Rached, Ana Diaz-Cives, Sophie Deguelte, Rami Rhaiem, Atoosa Khosravani, Amir Hossein Davarpanah Jazi, Reza Kianmanesh

**Affiliations:** 1 Surgery, Cabrol Hospital, CHU de Reims, Université de Reims Champagne Ardenne, Reims, FRA; 2 Surgery, Hazrat-E Fatemeh Hospital, Iran University of Medical Sciences, Tehran, IRN

**Keywords:** acute intestinal occlusion, internal hernia, mesenteric defect cloture, roux-y-gastric bypass, small bowel obstruction, surgical glue

## Abstract

Background and objective

Small bowel obstruction (SBO) with internal hernia (IH) is a life-threatening complication following minimally invasive Roux-en-Y gastric bypass (mRYGB). IH occurs frequently within the first two to three years after surgery, with an incidence ranging from 0.5% to 9%. Mesenteric defect closure (MDC) is consistently recommended. However, the technique remains controversial. Suturing, which is the most commonly used technique, can be risky due to mesenteric hemorrhage and hematoma. Glue application for MDC may offer an alternative. The objective of this observational study was to report the incidence of IH after the routine use of surgical glue for MDC during mRYGB.

Methods

We conducted an observational study to investigate this topic. From January 2020 to January 2022, a total of 170 consecutive patients who underwent mRYGB with surgical glue used for MDC were prospectively followed, and their data were analyzed.

Results

The mean age of the cohort was 43 ± 10 years. The mean BMI was 43 ± 3 kg/m². Laparoscopic RYGB was performed in 79% of patients, while robotic mRYGB accounted for 20%. The mean excess weight loss was 14 ± 16%. The median glue application time was eight minutes (range: 6-10 minutes), including application and polymerization time. The mean follow-up duration was 42 months (range: 17-71 months); 80% of patients had more than 24 months of follow-up. No clinically significant IH was observed during the follow-up period, while one patient underwent surgery for acute SBO without IH.

Conclusions

The preliminary results of MDC using surgical glue during mRYGB are promising in terms of feasibility and tolerance. The absence of IH observed during this follow-up is noteworthy but should be interpreted with caution due to the presence of potential biases. Further multi-institutional prospective studies are needed to gain deeper insights into the topic.

## Introduction

Minimally invasive Roux-en-Y gastric bypass (mRYGB) and sleeve gastrectomy are the two most commonly performed procedures in bariatric surgery worldwide [1]. Internal hernia (IH) is the most common and potentially life-threatening cause of small bowel obstruction (SBO) after mRYGB [2]. Its incidence ranges from 0.5% to 9% within the first two to three years, and may reach up to 14.4% during long-term follow-up [2-4]. SBO due to IH is associated with significant mortality rates: 3 to 5% in the absence of intestinal ischemia, and 30% in its presence [1-5].

Four potential mesenteric defects have been described. Among them, Petersen's space (PS) and the jejuno-jejunostomy mesenteric defect (JJD) are the most commonly reported [5]. Randomized clinical trials (RCTs) and meta-analyses recommend performing routine mesenteric defect closure (MDC), as it reduces the risk of IH from 8 to 9% to less than 2.5% [6-8]. However, there is no consensus regarding the closure technique [4,7,9]. While continuous non-absorbable barbed sutures are widely utilized, alternative techniques such as the use of clips, staples, meshes, and surgical glue have also been described [4,7,10-12].

## Materials and methods

Study population

Between January 2020 and January 2022, consecutive patients undergoing mRYGB, performed by a single surgeon (Dr. ADC) at Reims University Hospital (UH), France, were prospectively included in this single-center observational study. All surgical indications were validated by a multidisciplinary team at the local and regional UH Center of Specialized Obesity Surgery (CSO). All patients received written information about the surgical procedure and postoperative follow-up in their admission booklet, in accordance with institutional and national policies. The study was conducted in line with the principles of the Declaration of Helsinki (2024 revision), and all data were anonymized and handled in compliance with the General Data Protection Regulation (GDPR).

Surgical technique

**Figure 1:**
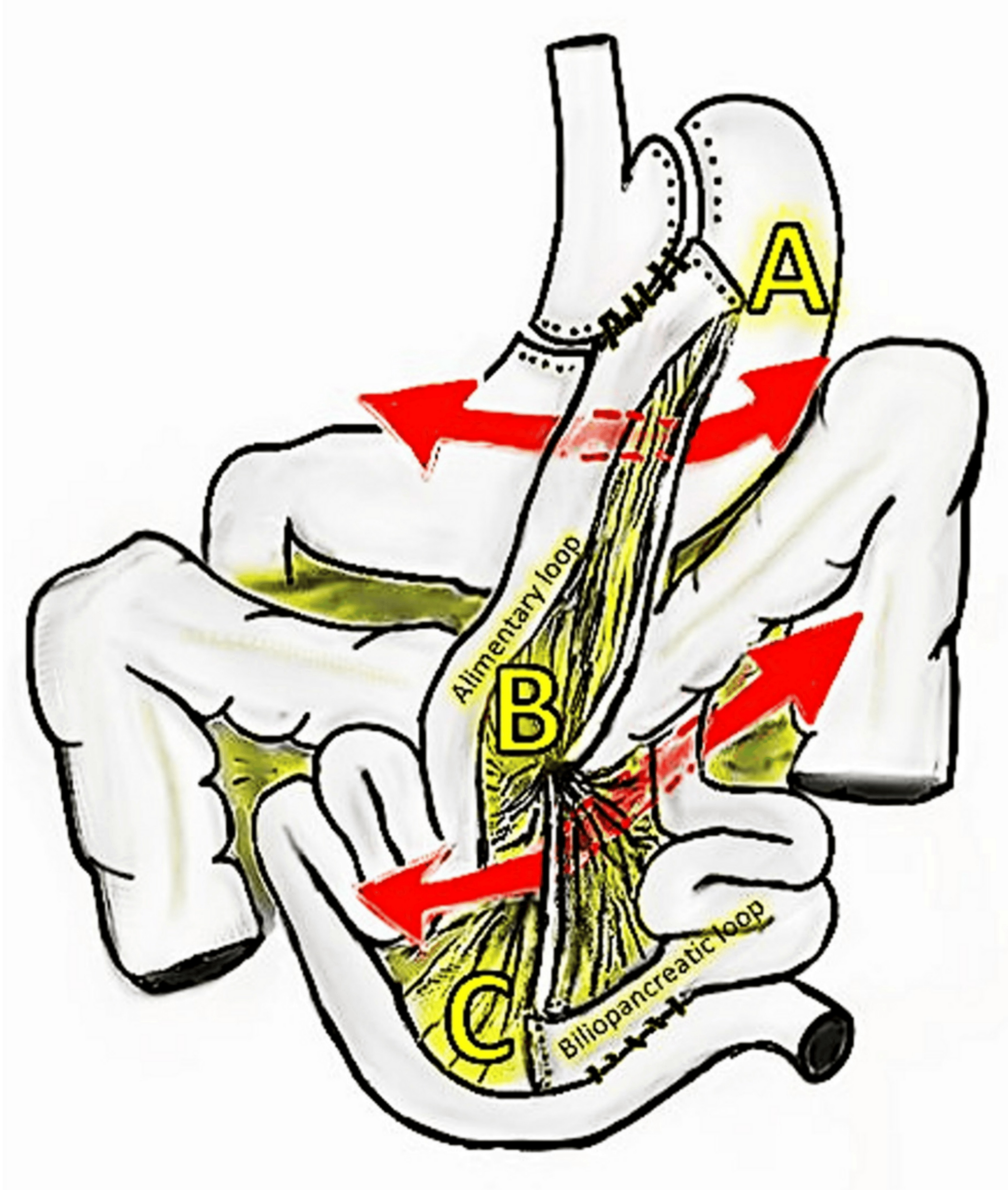
Presentation of PS and JJD Line A to B represents PS, and line B to C represents JJD. These mesenteric defects represent high-risk areas for internal hernia occurrence (red arrows) PS: Peterson’s space; JJD: jejunojejunal defect

Minimally invasive procedures include laparoscopic or robotic mRYGB. Primary procedures are performed on patients with no history of prior bariatric surgery. A standard mRYGB procedure was performed using the “modified Lönroth technique,” with a bilio-pancreatic limb of 90 cm and an alimentary limb of 150 cm in an antecolic position (Figure 1) [13]. The anterior surfaces of both gastrojejunal and Jejunojejunal mechanical anastomoses were closed with V-Lock® barbed sutures [14].

Glue application technique

**Figure 2:**
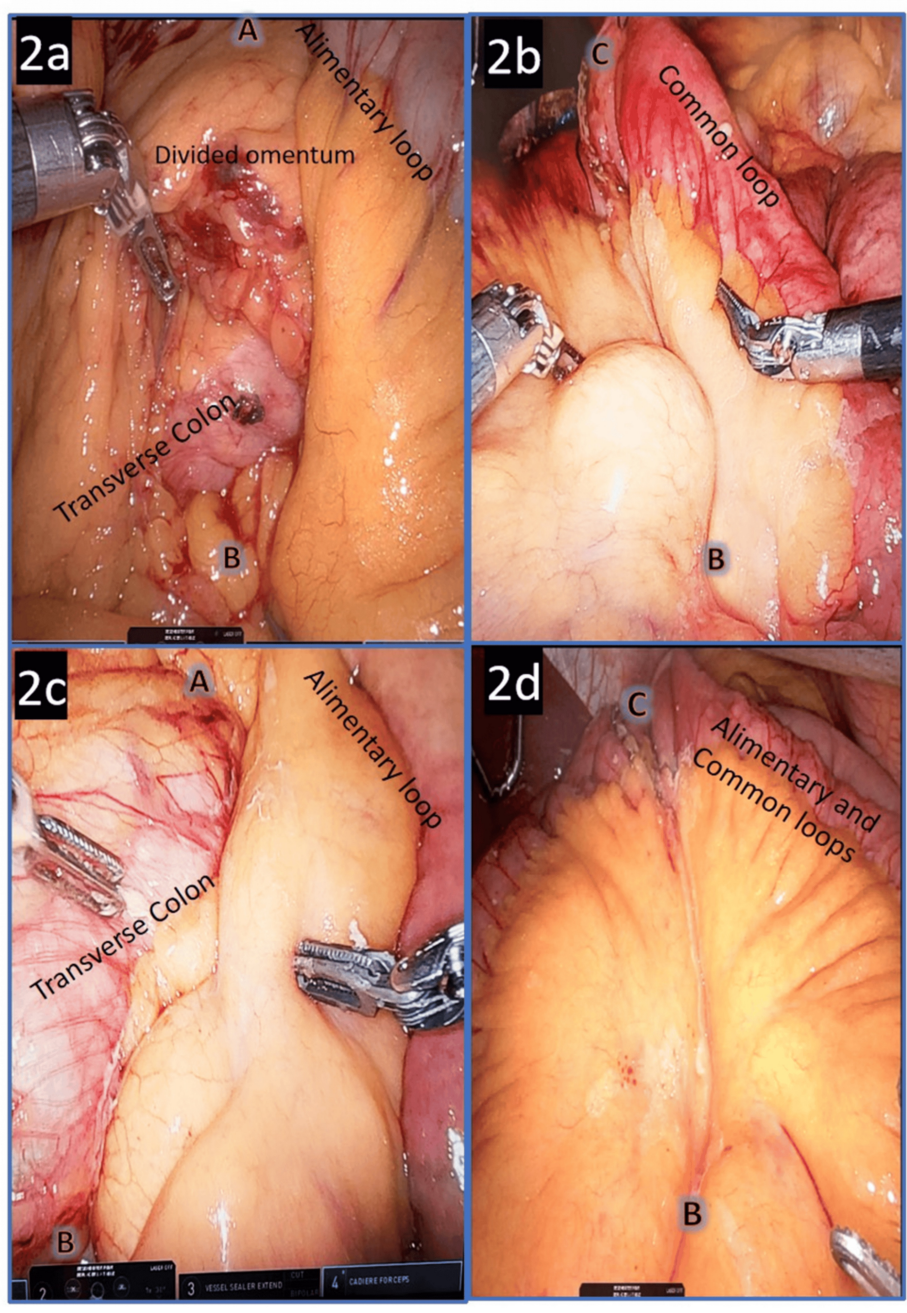
Intraoperative images of both PS and JJD before and after glue application After glue application (Figures 2b, 2c, 2d), a slight white line corresponding to the glue polymerization appeared. Glue was mainly applied to the anterior mesenteric side of the defects PS: Peterson’s space; JJD: jejunojejunal defect

MDC was performed by applying 1.5 mL of n-Hexyl-Cyanoacrylate surgical glue (Ifabond®) to both the PS and the JJD [11,15]. Glue was applied to the anterior mesenteric surfaces from point A to B for PS and from point B to C for JJD. Each mesenteric defect side was covered by the other side by at least 2 to 4 mm, as illustrated in Figures 1, 2. During the glue application, the surgeon applied the glue while the assistant delivered it gradually. Polymerization was observed by the appearance of a white edge. The quality of glue application was assessed by the surgical team (N = 3) and two theater nurses as follows: Grade 1 (G1) indicated poor or unsatisfactory closure, with non adherent mesenteric layers and a persistent visible gap; Grade 2 (G2) corresponded to partial or suboptimal closure, with some mesenteric adhesion but a remaining visible defect; and Grade 3 (G3) represented complete or optimal closure, with full mesenteric adhesion and no residual defect. If the glue application and or polymerization were not satisfactory (G2 or G1), a supplementary 1.5 mL of glue was applied, primarily on the posterior side of the mesenteric defect. No surgical drains were placed.

Postoperative evaluation

Before discharge, typically on postoperative day one, all patients underwent oral contrast-enhanced imaging using gastrografin in accordance with institutional protocol. Postoperative follow-up was conducted every six months for a minimum of two years, either at the UH or through affiliated regional centers. To complete the follow-up, all patients who had not returned to the UH for the past six months were contacted by phone. As the surveillance technique might underreport events, we recommended that all patients seek medical attention in the presence of any abdominal pain or persistent dry vomiting. Also, physicians were advised and made aware to perform emergency oral contrast gastrografin imaging for any patients who had undergone RYGB and presented with abdominal pain and or dry vomiting.

Statistical analyses

Qualitative values are expressed as N (percentages), and quantitative variables are expressed either as mean ± standard deviation (SD) or as median (range). *P*-values of less than 0.05, when reported, were considered significant. As the article is primarily observational and descriptive, no univariate or multivariate analyses or sampling methods were used.

## Results

All patients who underwent mRYGB during the study period were included consecutively without selection, resulting in a total of 170 patients being enrolled in the study. Baseline characteristics of the studied population are presented in Table 1. Primary mRYGB accounted for 79% of cases (Table 1). Robotic surgery was performed in 20% of patients. The median duration of the glue procedure was eight minutes (range: 6-10 minutes), including a median glue polymerization time of 61 seconds (range: 30-75 seconds). All glue-based MDCs were rated as G3 (optimal grade), except for one patient (graded G2) who received an extra 1.5 mL glue application on the posterior side of JJD. No intraoperative incident was reported.

**Table 1:** Main characteristics of the study population BMI: body mass index; RYGB: Roux-en-Y gastric bypass

Characteristic	Values
Gender, M/F	22/148
BMI before RYGB, kg/m^2^, mean (range)	43 (30-53)
Diabetes, n (%)	29 (17%)
Hypertension, n (%)	43 (25%)
Sleep apnea syndrome, n (%)	91 (54%)
Primary RYGB, n (%)	134 (79%)
Previous gastric binding - ablated before RYGB	23-16
Previous sleeve gastrectomy	13 (8%)

Overall outcomes, including postoperative complications, are presented in Table 2. The mean follow-up was 42 months (range: 17-71 months). The percentage of patients having a follow-up of more than two and three years was 80% and 41%, respectively. The overall rate of clinical and/or radiological SBO in this population was 0.6%, with no case of clinically relevant IH observed during the follow-up. One patient presented with SBO, three years after mRYGB with MDC by glue application. Emergency laparotomy revealed a tight, 1 cm jejuno-mesenteric adhesion with 20 cm of intestinal necrosis secondary to strangulation of the JJ anastomosis. Both the PS and JJD were intact, with reopening after three years of glue application. The localized tight adhesion was secondary to the remnant portion of the barbed suture of the JJ anastomoses. This patient had experienced important weight loss, with BMI decreasing from 48.9 preoperatively to 26.8 before the emergency surgery.

**Table 2:** Postoperative results (N = 170) ^*^Number of patients with one or more complications RYGB: Roux-en-Y gastric bypass; GSS: glue satisfaction score; GJ: gastrojejunal; JJ: Jejunojejunal; SBO: small bowel obstruction; IH: internal hernia; BMI: body mass index

Variables	Values
Laparoscopic, n (%)	136 (80%)
Robotic, n (%)	34 (20%)
Secondary RYGB, n (%)	36 (21%)
Glue application, n (%)	170 (100%)
GSS*: G1, G2, G3	0, 1, 169
Glue application time, minutes, median (range)	8 (4-10)
Glue polymerization time, seconds, median (range)	61 (30-75)
Postoperative complications^*^, n (%)	30 (18%)
Abdominal pain	15 (9%)
Dysphagia	3 (1.7%)
Vomiting	4 (2%)
Diarrhea	1 (0.5%)
GJ stenosis	1 (0.5%)
Fever	2 (1%)
GJ bleeding	1 (0.5%)
Melena (rectorrhagia)	2 (1%)
GJ fistula (mild)	2 (1%)
JJ fistula	1 (0.5%)
Incisional hernia	1 (0.5%)
SBO on adhesion	1 (0.5%)
SBO on IH	0
Cause of reoperations (revisions), n (%)	3 (1.7%)
JJ fistula	1 (0.5%)
Incisional hernia (at 4 months)	1 (0.5%)
Small bowel necrosis (at 4 years)	1 (0.5%)
Cholecystectomy (at 3 years)	1 (0.5%)
Salpingectomy, vaginal prolapse, cesarean	6 (3.5%)
Appendectomy, other (at 1 year)	2 (1%)
BMI post-RYGB, kg/m^2^, median (range)	29 (20-43)
Follow-up period, months, median (range)	42 (17-71)
Number of patients with more than 24 months of follow-up, n (%)	136 (80%)

## Discussion

The preliminary short-term results of this single-center observational study suggest that MDC using n-Hexyl-Cyanoacrylate surgical glue during mRYGB is a simple and reproducible technique with promising results. Although many biases are present in this study, the absence of IH due to the reopening of the mesenteric defects after a follow-up period of 42 months is encouraging.

Indeed, large studies have reported an incidence of IH of 2.5% at two to three years (range: 0.5-9%), reaching up to 14% after 13 years [3,7,10,13-15]. To reduce the rate of IH, the literature review strongly recommends routine closure of PS and JJD, yet the optimal MDC technique remains non-consensual and debated [2,6-8]. Running sutures are the most commonly used technique, while clips, staples, and meshes have been reported [2,4,7,14]. Still, this most commonly used technique, running sutures, may be associated with specific complications, especially in less specialized hands, such as mesenteric hemorrhage or hematomas, and does not eliminate the risk of kinking of the anastomosis and or defect reopening during follow-up [16-19]. The use of glue has been previously reported in a prospective non-randomized trial by Sina and Mouawad with promising results [11]. We therefore conducted an observational study to report our experience with the use of this glue technique.

IH might be due to the absence of complete closure initially and or reopening of the defects after closure, or both, especially after significant weight loss. The reopening mainly concerns the JJD. Indeed, the series by Vuagniaux et al. [2] reported a 5.8% IH rate after mRYGB, with the highest rate of IH through the JJD (almost three times more frequent than PS). Our results are in concordance with previously reported results showing that glue application for MDC during mRYGB can be safe, with promising short-term results [11]. Bruinsma et al. [9] found that 53% of patients undergoing reoperation had at least one mesenteric defect completely or partially open, despite initial MDC. In the present study, no clinical reopening was observed after 42 months.

Figure 1 shows the possible routes of intestinal entrapment through PS and JJD. It is important to note that intestinal entrapment may occur in both sides of PS and JJD. For example, the alimentary loop can pass through JJD from right to left side as well as from left to right side (see red arrows in Figure 1). This remains possible also for the bilio-pancreatic loop. In parallel, the use of antecolic alimentary loops seems to reduce the occurrence of IH after mRYGB and might contribute to better results [10]. As elongating the bilio-pancreatic loop is proposed in some centers (from 60 to more than 90-100 cm), this might mechanically generate quicker and more significant weight loss and induce larger JJD. The present series showed a median MDC by glue application time of eight minutes. This was longer than the five-minute reported duration of suturing of mesenteric defects in experienced hands; however, the reported time included the application and the polymerization of the glue [2,8]. 

Importantly, in this preliminary study, the only SBO requiring reoperation in our center was unrelated to JJD reopening three years after glue application, and at the time of reoperation, the surgeon observed a tight adhesion due to remnant barbed stump without MD reopening, as described in the literature [20]. An important technical point was the standardization of the glue application as described in the M&M section. Indeed, the two mesenteric loops' sides should lie on the other side so the glue could safely close the mesenteric defects, including the intestinal surfaces (Figures 1, 2). 

This study has several limitations. This was a single-center, single-surgeon, prospective observational study with no control group. The follow-up was short (42 months). However, the previously reported study using the same glue for MDC was prospective and non-randomized, with a follow-up that was half as long (28 months) [11]. All these limitations should be considered when interpreting the results with great caution. Nevertheless, we believe that the consistency of outcomes across 170 consecutive cases, even over a short-term period, might suggest that glue-based MDC warrants further investigation. The present study, together with the previously reported series using the same type of glue for MDC after mRYGB, suggests a possible basis for conducting multicenter studies and even RCTs. Such studies might better assess not only IH rates, but also complication profiles, procedural efficiency, learning curves, and long-term durability.

## Conclusions

IH is a life-threatening complication after mRYGB. The present series reported interesting preliminary results after the routine use of glue for MDC during mRYGB, with no IH observed after 42 months of follow-up. However, the results should be interpreted with great caution due to multiple biases, including the absence of a control group and the short follow-up period. We believe that glue application for MDC during mRYGB might be a valid alternative technique. Further large, multicenter, and or prospective comparative studies are needed.
